# Supplementary material for: Local avalanche photodetectors driven by lightning-rod effect and surface plasmon excitations
Source: Nat Commun. 2025 Dec 3;17:76. doi: 10.1038/s41467-025-66790-w (PMC12769478; doi:10.1038/s41467-025-66790-w)
Supplement: Supplementary file 1 — Supplementary Information File [file 41467_2025_66790_MOESM1_ESM.pdf]

Supplementary Information

Local Avalanche Photodetectors Driven by Lightning-rod Effect and Surface Plasmon Excitations

Zhao Fu[1,2], Jia Liu[1], Meng Yuan[1], Jiafa Cai[1,3], Rongdun Hong[1,3], Xiaping Chen[1,3], Dingqu Lin[1,3], Shaoxiong Wu[1,3], Yuning Zhang[1], Zhengyun Wu[1,3], Zhanwei Shen[4]*, Zhijie Wang[4]*, Jicheng Wang[5]*, Mingkun Zhang[1,6]*,Zhilin Yang[1]*, Deyi Fu[1]*, Feng Zhang[1,3]*, Rong Zhang[1]*

zwshen@semi.ac.cn, wangzj@semi.ac.cn, jcwang@jiangnan.edu.cn, mkzhang@xmu.edu.cn, zlyang@xmu.edu.cn, dyfu@xmu.edu.cn, fzhang@xmu.edu.cn, rzhangxmu@xmu.edu.cn

[1]Department of physics, Xiamen university, Fujian, 361005, P. R. China

[2]College of Electrical Engineering, Tongling university, Anhui, 244061, P. R. China

[3]Jiujiang Research Institute of Xiamen University, Jiangxi, 332000, P. R. China.

[4]Laboratory of Solid-State Optoelectronics Information Technology, Institute of Semiconductors, Chinese Academy of Sciences, Beijing, 100083, P. R. China

[5]School of Science, Jiangnan University, Jiangsu, 214122, China

[6]The Higher Educational Key Laboratory of Flexible Manufacturing Equipment Integration of Fujian Province, Xiamen Institute of Technology, Fujian, 361005, P. R. China

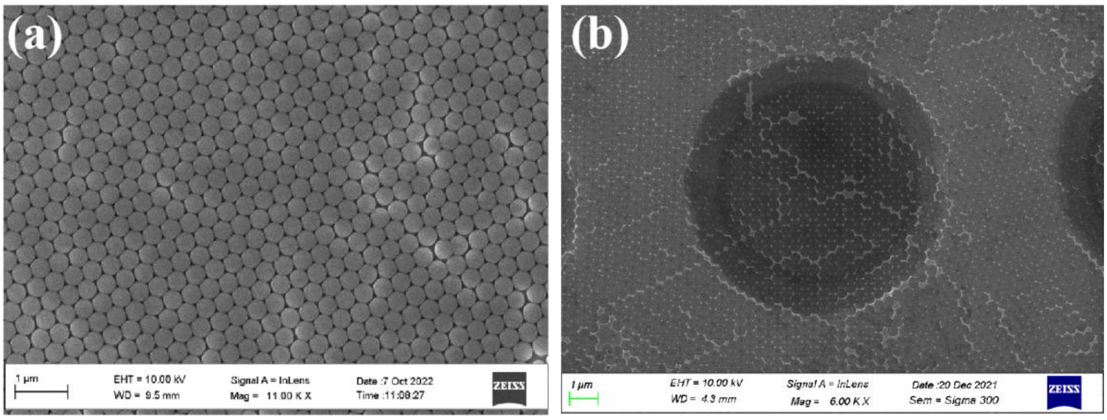

Fig. S1. (a) The SEM image of PS microspheres template covered on the surface of the devices; (b) Section morphology and magnified view of Al NTs arrays in the MHs.

Equation: The detectivity of device was calculated according to the responsivity(R), dark current (I_d) and photosensitive area (A) by the formula (1).

$$D^{*}=R\times\sqrt{\frac{A}{2qI_d}} \quad (1)$$

Gauss's theorem equation.

$$\theta_e=\oiint \mathbf{E}\cdot d\mathbf{S}=\frac{\Sigma Q}{\varepsilon_0} \quad (2)$$

Where **E** and **S** represent the electric field and the closed Gaussian surface, respectively. Q is the amount of charge in a closed Gaussian surface.

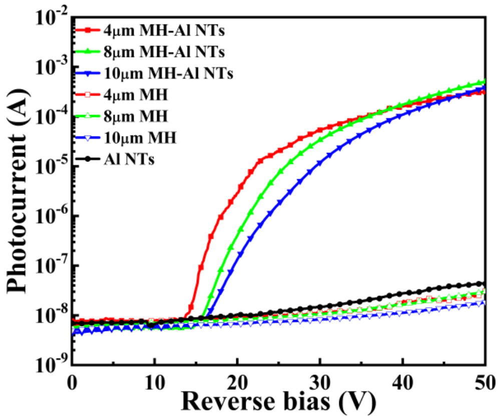

Fig. S2. The comparison of photocurrent for the MH-PDs with and without Al NTs for different diameters MHs (4 μm, 8 μm and 10 μm) and PDs with Al NTs.

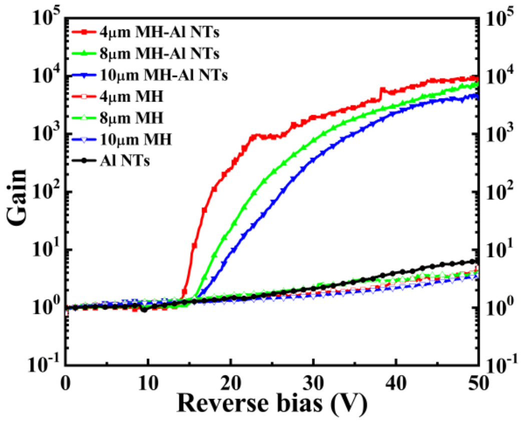

Fig. S3. The comparison of gain for the MH-PDs with and without Al NTs for different diameters MHs (4 μm, 8 μm and 10 μm) and PDs with Al NTs.

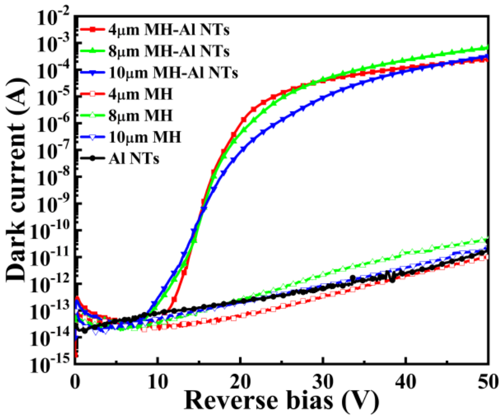

Fig. S4. The comparison of dark current for the MH-PDs with and without Al NTs for different diameters MHs (4 μm, 8 μm and 10 μm) and PDs with Al NTs.

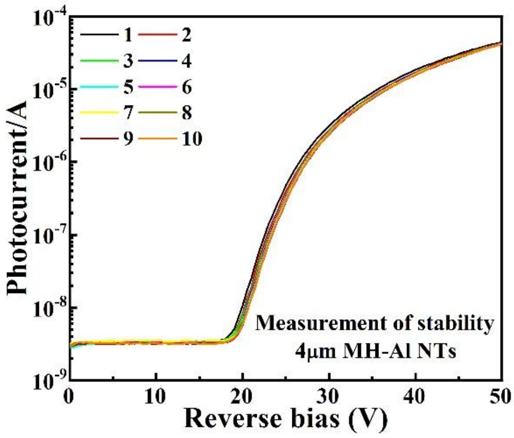

Fig. S5. The photocurrent–voltage curves by multiple measurements.

The detectivity of the device shown in Figure 3(c) of the main text is calculated based on the responsivity from the Supplementary Material's Fig. S6 and the dark current from Fig. S4, with the specific calculation method and numerical discussion as follows:

The specific expression for normalized detectivity is provided in the supplement $D^{*}=R\times\sqrt{\frac{A}{2qI_d}}$, which represents the photo-detection capability of the optoelectronic device and is correlated with the device's responsivity, dark current, and active area. In the "Design and Experiment" section of the article, the single square pixel is 200 μm × 200 μm as shown in Figure 1(a), and the dark current I-V curve of the device is shown in Figure S4 in the supplementary material. Moreover, the spectral response is presented below and has already been incorporated into the supplementary

material, which includes the device's responsivity and quantum efficiency. In summary, with the photosensitive area, dark current, and responsivity of the device, the detectivity can be obtained in Figures 2(c) and (d).

For example, for the 4 μm device at 280 nm at 10 V, its responsivity R is 0.129 A/W, the dark current I_d is 5.0×10^{-14} A, and the device area is 200 μm × 200 μm. Thus the normalized detectivity D^* is 2.0×10^{13} Jones. The specific calculation procedure is demonstrated by the following formula:

$$R=\frac{I_{ph}}{P_{opt}}=\frac{7.6\times10^{-9}\text{ A}}{5.89\times10^{-8}\text{ W}}=0.129\text{ A/W} \tag{3}$$

$$D^*=R\times\sqrt{\frac{A}{2qI_d}}=0.129\text{ A/W}\cdot\sqrt{\frac{0.02\text{ cm}\cdot0.02\text{ cm}}{2\times1.6\times10^{-19}\text{ C}\cdot5.0\times10^{-14}\text{ A}}}=2.0\times10^{13}\text{ Jones} \tag{4}$$

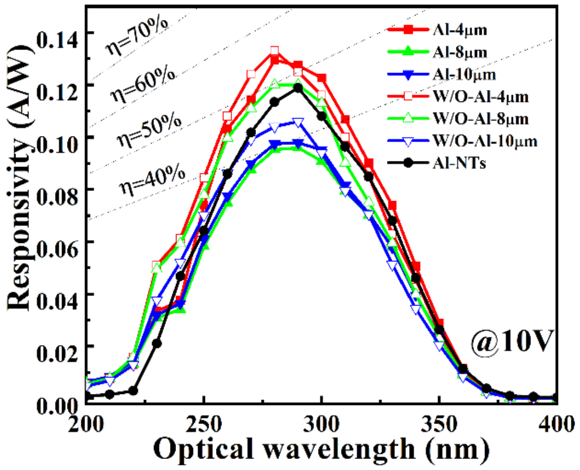

Fig. S6. Spectral response and quantum efficiency graphs of APD devices with MHs without Al NTs, with MHs with Al NTs, and without MHs with Al NTs, under 10 V reverse bias.

The avalanche noise level depends on the ionization rate ratio (α_N/α_P) and the avalanche gain (M). For the case of hole injection, the noise level can be expressed as :

$$F=M[1-(1-k)(\frac{M-1}{M})^2]\approx kM+(2-\frac{1}{M})(1-k) \tag{5}$$

where $k=\alpha_N/\alpha_P$, which remains constant throughout the avalanche region. For SiC material, the impact ionization coefficients of electrons and holes can be expressed as:

$$\alpha_N(E)=1.69\times10^6\text{ cm}^{-1}\exp[-(\frac{9.96\times10^6\text{ V/cm}}{E})^{1.6}] \quad \text{(electron)} \tag{6}$$

$$\alpha_P(E)=3.32\times10^6\text{ cm}^{-1}\exp[-(\frac{1.07\times10^7\text{ V/cm}}{E})^{1.1}] \quad \text{(hole)} \tag{7}$$

where E represents the electric field intensity.

The avalanche electric field intensity in conventional SiC APDs is typically 3 MV/cm. By substituting E=3 MV/cm into the equations above, we calculate that $k \approx 0.032$. Consequently, the noise level in conventional devices can be expressed as:

$$F=0.032M-0.968/M+1.936 \qquad (8)$$

For comparison, the local avalanche electric field intensity in our fabricated device is 1.5 MV/cm under a reverse bias of 15 V as shown in Fig. S8. We therefore simulated the local avalanche electric field intensity under different reverse bias conditions. Since the gain is related to the reverse bias (as shown in Fig. 2a), Fig. S7 was obtained by curve fitting.

The results show that the noise level increases with avalanche gain. The fabricated device exhibits a lower noise level compared to the device without MH-Al NTs, owing to localized avalanche effects.

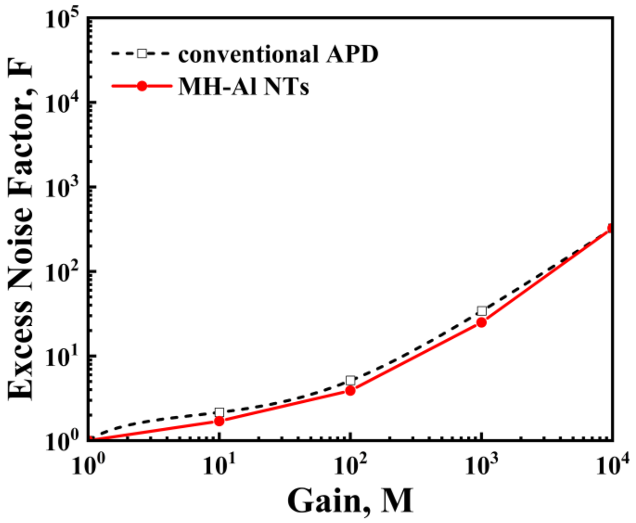

Fig. S7. Comparison of excess noise factor in the device without MH-Al NTs and the fabricated device.

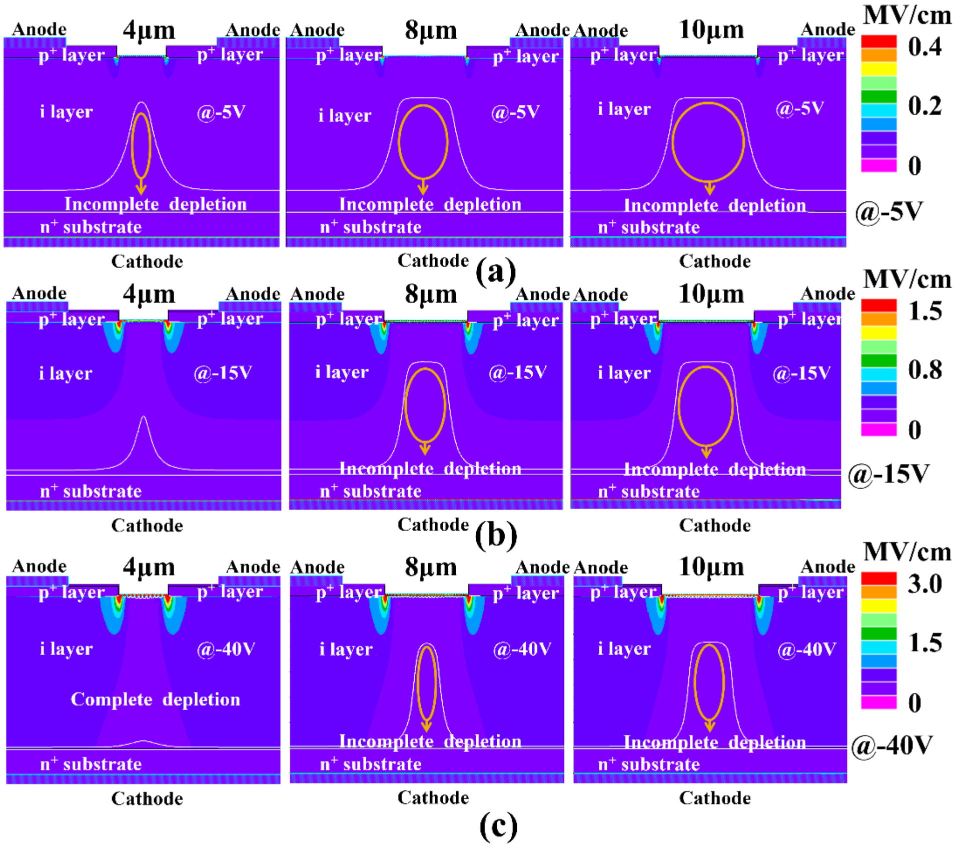

Fig. S8. The internal electric field intensity of the devices with MH (4 μm, 8 μm and 10 μm) and Al NTs under the charge accumulation effect at the tips of Al NTs was simulated by applying (a) 5 V, (b) 15 V, (c) 40 V reverse bias.

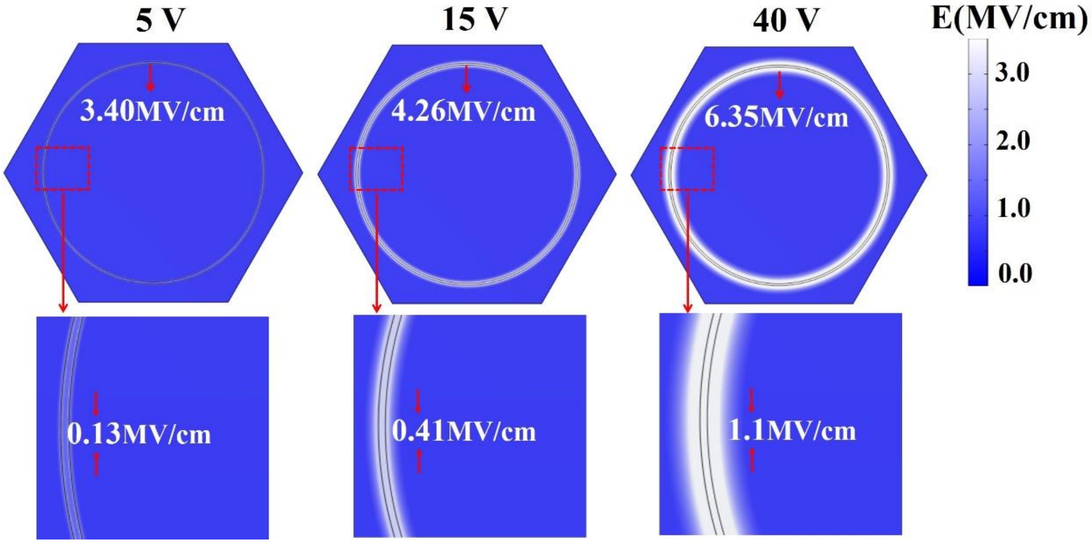

Fig. S9. The electric field intensity of the devices without Al NTs was simulated at different reverse bias (5 V、15 V and 40 V).

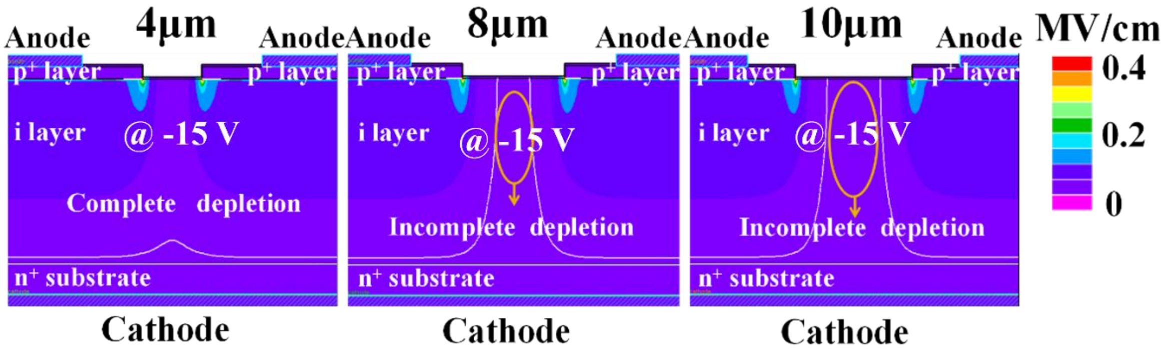

Fig. S10. The internal electric field intensity of the devices without Al NTs by applying 15 V reverse bias.

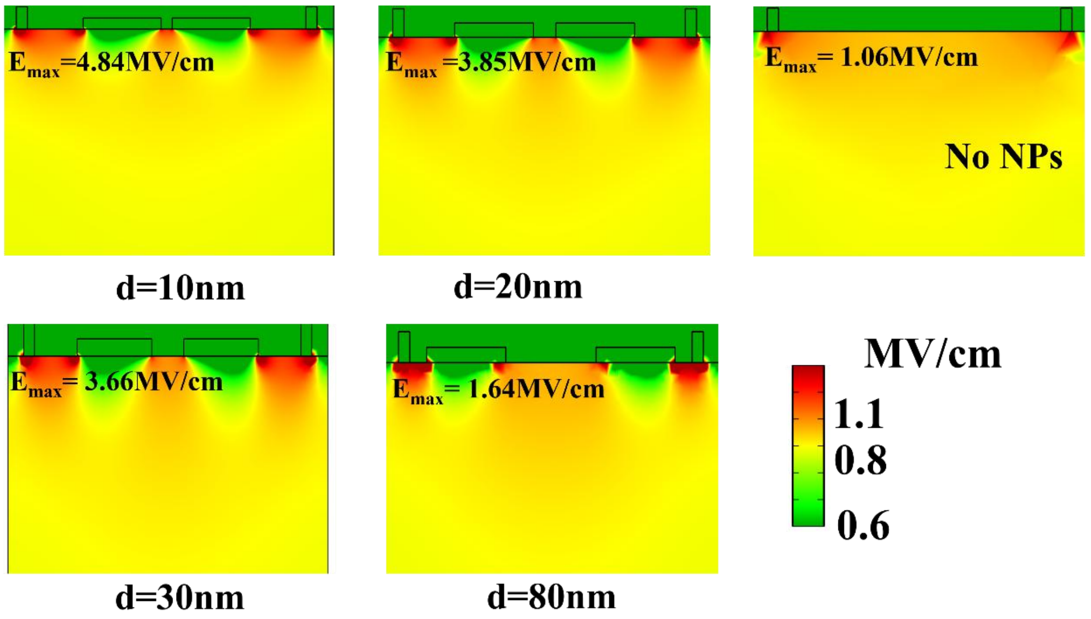

Fig. S11. Simulation diagrams of the electric field inside the device under different inter spacing(10 nm、20 nm、30 nm and 80 nm) conditions of Al NTs by COMSOL. When the inter-spacing between Al NTs is 10 nm, 20 nm, 30 nm, 80 nm, and for the device without Al NTs, the maximum electric fields within the devices are 4.84 MV/cm, 3.85 MV/cm, 3.66 MV/cm, 1.64 MV/cm, and 1.06 MV/cm, respectively.

Below Figure 4(d) illustrates a schematic of the localized avalanche phenomenon, where it can be observed that carrier collision ionization occurs on both conduction band and valence band. This indicates that avalanche takes place at the edges of the MHs, whereas the remaining regions remain quiet. Figure S11 depicts the effect of varying inter-spacing between Al NTs on the internal electric field of the device simulated by COMSOL. On one hand, it is evident that devices with Al NTs exhibit a

significantly stronger internal electric field compared to those without Al, with the maximum field strength reaching 4.84 MV/cm at a spacing of 10 nm. Additionally, it is observed that the maximum internal electric field within the device increase as the spacing between Al NTs decrease. This increase is primarily due to the enhanced coupling effect that occurs between closely spaced Al NTs, which results in a stronger electric field.

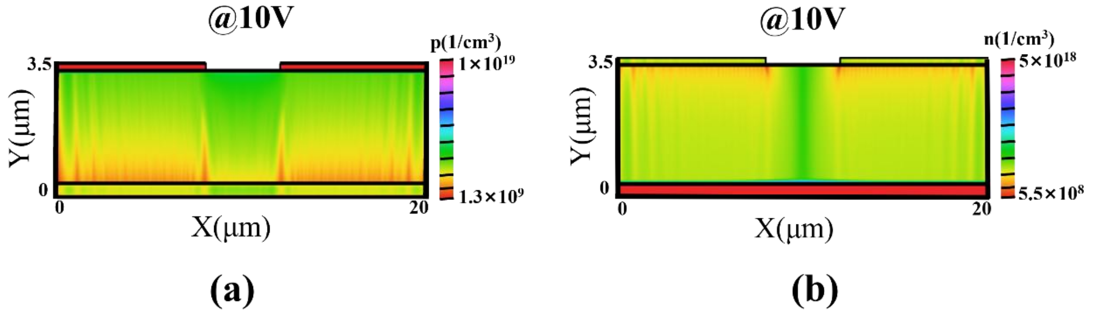

Fig. S12. (a) Under a 10 V reverse bias, the distribution of hole concentration within the device. (b) under a 10 V reverse bias, the distribution of electron concentration within the device.

To better comprehend the carrier concentration within the device, we simulated the spatial distributions of hole and electron concentrations, as illustrated in Fig. S12. The results reveal that in regions with high electric fields, the depletion of the electric field is more pronounced, leading to an increased electron concentration. Furthermore, the contribution of incident photons to carrier generation and field enhancement can be analyzed through photocurrent and quantum efficiency. To elaborate, the photocurrent I_{ph} is measured as $I_{ph}=7.6\times10^{-9}$ A (@10 V/280 nm/5.89×10^{-8} W), with the elementary charge $q = 1.6\times10^{-19}$ C, and a quantum efficiency $\eta= 57.2\%$. Based on these parameters, we can perform the following calculations. First, we calculate the number of photogenerated carriers produced within 1 second: $N = I_{ph} * t / q = 4.75\times10^{10}$, which corresponds to 47.5 billion electron-hole pairs. Subsequently, we determine the number of incident photons M, where $M = N / \eta = 8.3 \times 10^{10}$. In summary, 42.8% of the incident photons do not contribute to the generation of photogenerated carriers. This proportion accounts for light reflection, carrier recombination due to defects, and field enhancement. Reflection spectrum testing, as shown in Fig. S13, indicates that the reflectivity at 280 nm is approximately 16.0%. Therefore, the photons contributing to

field enhancement account for less than 26.8%, corresponding to fewer than 2.3×10^{10} photons.

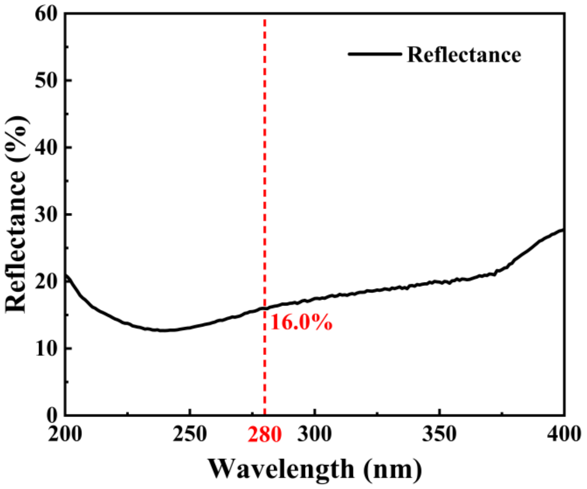

Fig. S13. The reflection spectrum of the device with MH and Al NTs.

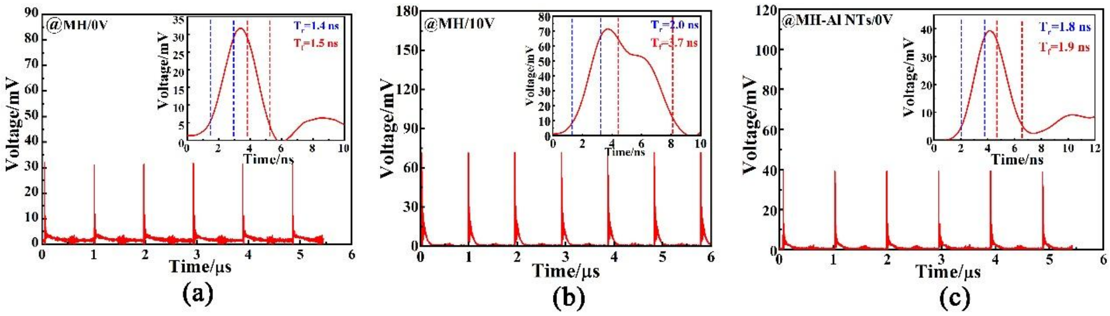

Fig. S14. The response times of the devices with MH at (a) 0 V, (b) 10 V, and (c) Al NTs at 0 V. The insets are magnified single impulses.

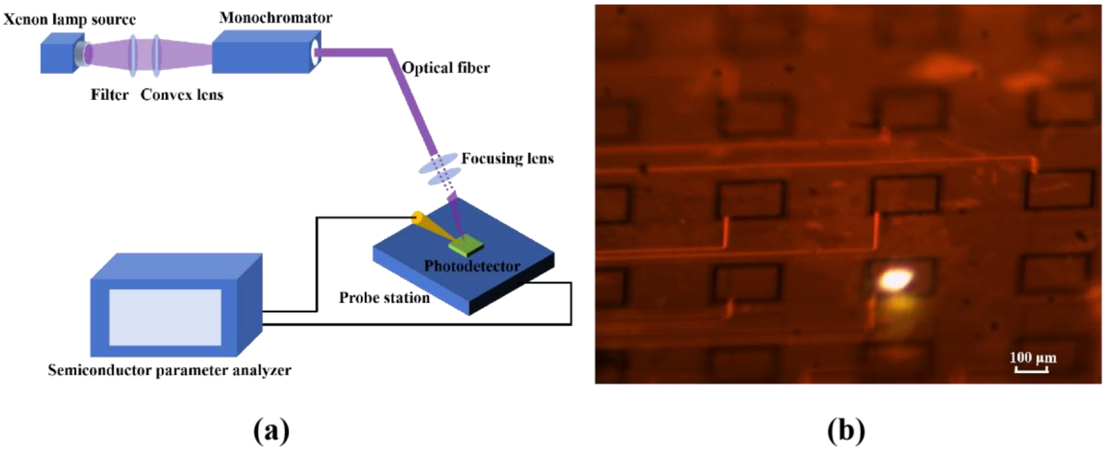

Fig. S15. (a) Schematic diagram of the device performance measurement system. (b) Focused light spot on the device photosensitive area (optical microscope image)

In the experiment, a light source with 5.89×10^{-8} W total power(@280 nm) was focused on a circular area with a diameter of approximately 120 μm, resulting in a power 5.89×10^{-8} W incident over the 200 μm × 200 μm pixel area.

For instance, at a wavelength of 280 nm, the Si-222 standard photodetector exhibits a spectral responsivity of 0.0958 A/W. With an applied bias, a photocurrent of 5.64×10^{-9} A was measured, yielding a calibrated optical power of 5.89×10^{-8} W. Subsequently, the fabricated devices were tested. As shown in Figure. S15. (b), the light from the xenon lamp was coupled through the optical fibre and focused into a circular spot with a diameter of approximately 120 μm. This confirms that the total optical power of 58.9 nW was illuminated onto the photosensitive area of the device. After aligning the light spot, the monochromator was set to a wavelength of 280 nm for the measurement, from which the photocurrent-voltage (I-V) curve of the device was obtained.
